# Supplementary material for: From sequence to enzyme mechanism using multi-label machine learning
Source: BMC Bioinformatics. 2014 May 19;15:150. doi: 10.1186/1471-2105-15-150 (PMC4229970; doi:10.1186/1471-2105-15-150)
Supplement: Additional file 2 — Java code of ml2db. Additional file ml2db_code.tar.gz contains the Java source code to run the multi-label machine learning experiments and save the results to database. The code’s Javadoc is included. [file 1471-2105-15-150-S2.zip › additional file 2/ml2db/ml2db/doc/main/ml2db/mulan/learn/crosseval/Folds.html]

Folds


JavaScript is disabled on your browser.


- Overview
- Package
- Class
- Use
- Tree
- Deprecated
- Index
- Help

- Prev Class
- Next Class

- Frames
- No Frames

- All Classes

- Summary:
- Nested |
- Field |
- Constr |
- Method

- Detail:
- Field |
- Constr |
- Method


main.ml2db.mulan.learn.crosseval

## Class Folds

- java.lang.Object
- - main.ml2db.mulan.learn.crosseval.Folds

- Direct Known Subclasses:
  :   StratifiedFolds

  ---

    

  ```
  public class Folds
  extends java.lang.Object
  ```

  Stores and delivers folds for cross validation

  Version:
  :   22 Mar 2013

  Author:
  :   ldf

- - ### Constructor Summary

    Constructors

    Constructor and Description

    `Folds(IdMultiLabelInstances instances,
    int numFolds)`
  - ### Method Summary

    Methods

    | Modifier and Type | Method and Description |
    | --- | --- |
    | `int` | `getNumberOfInstances()` |
    | `IdMultiLabelInstances` | `getOriginalSet()` |
    | `mulan.data.MultiLabelInstances[]` | `getTrainTestFolds(int i)` Get the train and test data for crossevaluation fold i |

    - ### Methods inherited from class java.lang.Object

      `equals, getClass, hashCode, notify, notifyAll, toString, wait, wait, wait`

- - ### Constructor Detail


    - #### Folds

      ```
      public Folds(IdMultiLabelInstances instances,
           int numFolds)
      ```
  - ### Method Detail


    - #### getNumberOfInstances

      ```
      public int getNumberOfInstances()
      ```


    - #### getOriginalSet

      ```
      public IdMultiLabelInstances getOriginalSet()
      ```


    - #### getTrainTestFolds

      ```
      public mulan.data.MultiLabelInstances[] getTrainTestFolds(int i)
      ```

      Get the train and test data for crossevaluation fold i

      Parameters:
      :   `i` - the fold number
      :   `random` - a random seed

      Returns:
      :   the train (index 0) and test (index 1) set


- Overview
- Package
- Class
- Use
- Tree
- Deprecated
- Index
- Help

- Prev Class
- Next Class

- Frames
- No Frames

- All Classes

- Summary:
- Nested |
- Field |
- Constr |
- Method

- Detail:
- Field |
- Constr |
- Method
